# Quantitative real-time PCR detection of Porphyromonas gingivalis and Filifactor alocis in peri-implantitis

**DOI:** 10.1099/jmm.0.002091

**Published:** 2025-11-12

**Authors:** Ioannis Fragkioudakis, Georgios Konstantopoulos, Christine Kottaridi, Leonidas Batas, Dimitra Sakellari

**Affiliations:** 1 Department of Periodontology and Implant Biology, School of Dentistry, Aristotle University of Thessaloniki, Thessaloniki, Greece; 2 Department of Genetics, General Microbiology Laboratory, Development and Molecular Biology, School of Biology, Aristotle University of Thessaloniki, Thessaloniki, Greece

**Keywords:** biofilm, *Filifactor alocis*, peri-implantitis, peri-implant inflammation, *Porphyromonas gingivalis*, quantitative real-time PCR

## Abstract

**Introduction.** Peri-implantitis is a prevalent and challenging complication in implant dentistry, primarily induced by biofilm-associated pathogens. Among these, *Porphyromonas gingivalis* and *Filifactor alocis* have emerged as key contributors, with evidence suggesting their potential synergistic role in exacerbating peri-implant inflammation and tissue destruction.

**Hypothesis/Gap Statement.** While *P. gingivalis* is a well-characterized periopathogen, the specific role of *F. alocis*, alone or in combination with *P. gingivalis*, in peri-implantitis remains underexplored. This study addresses the gap in quantifying their presence in diseased versus healthy peri-implant sites.

**Aim.** To assess the prevalence and microbial load of *P. gingivalis* and *F. alocis* in peri-implantitis and healthy peri-implant sites using quantitative real-time PCR (qPCR) and to investigate their correlation with clinical parameters.

**Methodology.** This cross-sectional study included 110 participants: 52 diagnosed with peri-implantitis and 58 with healthy peri-implant tissues. Clinical examination recorded probing depth (PD), clinical attachment level (CAL) and bleeding on probing (BOP). Submucosal biofilm samples were collected and analysed using species-specific qPCR. Statistical analysis employed the Mann–Whitney *U* test for intergroup comparisons and Spearman’s rank correlation for associations between microbial levels and clinical indices.

**Results.** Both *P. gingivalis* and *F. alocis* were significantly elevated in peri-implantitis sites compared to healthy controls. Mean *P. gingivalis* levels were 4.80×10⁶ ± 4.78×10⁶ copies µl^−1^ in peri-implantitis and 2.09×10³ ± 1.26×10³ copies µl^−1^ in healthy sites (*P*<0.001). *F. alocis* levels averaged 4.58×10⁵ ± 3.40×10⁵ copies µl^−1^ in peri-implantitis and 2.45×10³ ± 1.64×10³ copies µl^−1^ in healthy sites (*P*<0.001). *P. gingivalis* showed strong positive correlations with PD, CAL and BOP, while *F. alocis* correlated moderately with PD and CAL but not significantly with BOP.

**Conclusion.** The significant elevation of *P. gingivalis* and *F. alocis* in peri-implantitis supports their potential synergistic involvement in disease pathogenesis. These findings underscore the need for antimicrobial strategies that target both organisms and disrupt their cooperative biofilm behaviour. Further research should clarify their pathogenic interplay and inform the development of precise therapeutic interventions.

## Data Summary

The data that support the findings of this study are openly available.

## Introduction

Peri-implantitis is an inflammatory condition involving the mucosa and supporting bone around osseointegrated dental implants, characterized by increased probing depths, bleeding on probing and radiographic bone loss [1]. This condition represents a major complication in implant dentistry, with prevalence rates ranging from 10 to 47% [2]. This variability can be attributed to factors such as differences in diagnostic criteria, population diversity and study methodologies [3,4]. If left untreated, peri-implantitis can lead to implant failure. Despite advances in implant technology, surgical techniques and primordial care protocols, peri-implantitis remains a significant clinical challenge, highlighting the need for a deeper understanding of the microbial aetiology and prevention underlying this disease [3,5, 6].

Among the micro-organisms implicated in peri-implantitis, *Porphyromonas gingivalis *has long been recognized as a critical periodontal pathogen [7]. It is a member of the ‘red complex’, a group of bacteria strongly associated with periodontitis and peri-implantitis [8,10]. *P. gingivalis* possesses multiple virulence factors, including fimbriae, capsules and gingipains, which allow it to invade host tissues, evade immune responses and contribute to tissue destruction through the modulation of host immune responses [7,11]. Its role in biofilm formation and its association with clinical parameters, such as probing depth and attachment loss, underscore its significance in the progression of peri-implant disease. These factors not only allow persistence and evasion but also actively modulate host immunity, promoting a dysbiotic and inflammatory peri-implant environment. Similarly, *F. alocis* is known to induce cytokine release via TLR2 signalling and resist oxidative stress, contributing to biofilm resilience and chronic inflammation [12,13].

Recent research has highlighted the presence of *Filifactor alocis *as an emerging pathogen in periodontal and peri-implant diseases [12]. Unlike many Gram-negative pathogens, *F. alocis* is a Gram-positive anaerobic bacterial species that exhibits unique traits, including resistance to oxidative stress and the ability to thrive in inflamed environments [12]. These characteristics allow it to persist within the biofilm of peri-implant pockets, contributing to chronic inflammation and tissue damage [12,14].

A notable aspect of *F. alocis* is its ability to synergize with *P. gingivalis* [12]. Studies have demonstrated that *P. gingivalis* facilitates the colonization of *F. alocis* by altering the local environment, creating conditions favourable for its growth [15]. In turn, *F. alocis* enhances the inflammatory response through immune modulation and oxidative stress resistance. Together, these two species form biofilms with increased virulence compared to when they are cultured individually. This cooperation underscores the importance of understanding their combined role in peri-implantitis to enforce the development of more effective treatment strategies [12].

This study aims to evaluate the prevalence and levels of *P. gingivalis* and *F. alocis* in peri-implantitis sites compared to healthy peri-implant sites using quantitative real-time PCR (qPCR). By focusing on their microbial synergy and specific contributions to peri-implant inflammation, this research seeks to inform targeted therapeutic strategies for managing peri-implantitis and improving patient outcomes.

## Methods

This study was designed as a cross-sectional investigation. This cross-sectional study was conducted at the Department of Periodontology and Implant Biology, Aristotle University of Thessaloniki. All participants were recruited from the Department of Periodontology and Implant Biology at the School of Dentistry, Aristotle University of Thessaloniki, Greece. According to the 2018 classification criteria, patients were classified into two groups: those with peri-implantitis and those with healthy implants or peri-implant mucositis [1]. The healthy/mucositis group included individuals with either healthy peri-implant tissues or signs of peri-implant mucositis, with no radiographic bone loss or clinical indicators of peri-implantitis. The Ethical Committee of the School of Dentistry, Aristotle University of Thessaloniki (115/25 May 2021), approved the study, which was registered in the ClinicalTrials.gov database under ID: NCT05711407.

### Study timeline

The study timeline included two appointments and subsequent laboratory analyses. Patients were diagnosed and recruited during the first appointment based on inclusion criteria, followed by a comprehensive clinical examination. The study took place between January 2023 and March 2024. A second appointment was scheduled 1 week later for biological sample collection. All samples were gathered between 8 : 00 AM and 10 : 00 AM to minimize diurnal fluctuations in microbial levels, and participants were instructed to fast for at least 8 h before sampling. Patients were also asked not to brush their teeth on the morning of sampling to avoid disturbing the biofilm.

### Participant inclusion criteria and sample size

Participants were selected based on predefined inclusion criteria. Eligible individuals had at least one implant loaded for over a year and were systemically healthy, which is defined as those without any known chronic systemic conditions, such as diabetes mellitus, cardiovascular disease, autoimmune disorders or other diseases that could influence periodontal or peri-implant health. They were either periodontally healthy or demonstrated stable periodontal disease as per Lang and Bartold [16]. Patients who had taken antibiotics in the last 6 months were excluded, while smokers were permitted to participate. All participants provided informed consent before enrolling in the study. The sample size was determined through a power analysis to detect significant differences in the relative abundance of key periodontal pathogens between the peri-implantitis and healthy implant groups. The analysis indicated that a minimum of 54 participants per group was necessary to achieve 80% power at a 5% significance level, using data from previous research on * P. gingivalis* levels in peri-implantitis [17].

### Clinical examination

Clinical parameters were recorded during the examination phase. The clinical examination included bleeding on probing (BOP), which was noted as present (+) or absent (−), observed 30 s after a periodontal probe was inserted into the peri-implant pocket and expressed as a percentage. Probing depth (PD) was measured from the mucosal margin to the base of the peri-implant pocket. Recession was recorded as the distance from the shoulder of the prosthetic crown to the mucosal margin, while clinical attachment level (CAL) represented the distance from the shoulder of the prosthetic crown to the base of the sulcus or peri-implant pocket. All measurements were made at six sites per implant using a 15-mm scale periodontal probe (Hu-Friedy® CP-12, #30), graded in 1 mm increments.

All examinations were conducted by the same examiner (I.F.), and intra-examiner reproducibility was assessed during two calibration sessions. The calibration sessions, conducted 2 weeks apart, involved repeated measurements on a sample of ten patients to ensure consistency across time points. The intra-examiner agreement, determined using the intraclass correlation coefficient, showed an agreement of 0.93 (95% Confidense Interval (CI) 0.89 to 0.96), reflecting high reliability.

### Sample collection

For microbiological sampling, each implant site was isolated using cotton rolls, and supragingival and marginal plaque were carefully removed to minimize contamination. Biofilm samples were then obtained from the deepest peri-implant sulcus or pocket by inserting three sterile endodontic paper points (no. 30) for 10 s. The paper points were immediately placed into sterile 1.5-ml microcentrifuge tubes and stored at −80 °C until analysis. The same standardized sampling protocol was applied to both study groups. In each participant, the deepest site was selected regardless of whether the site was clinically healthy, exhibited mucositis or showed signs of peri-implantitis. To improve sample accessibility and quality, prosthesis removal was performed whenever feasible in both groups.

### DNA extraction and qPCR analysis

DNA extraction was performed using the ZymoBIOMICS™ DNA Miniprep Kit (Zymo Research, USA), following the manufacturer’s instructions. To monitor extraction efficiency and control for inhibition, 4 µl of internal extraction control DNA was added to the lysis buffer of each sample.

qPCR was used to detect and quantify *P. gingivalis* and *F. alocis*. For *F. alocis*, the AccuPower® *F. alocis* Real-Time PCR Kit (Bioneer Corporation, South Korea) was used, which targets the 16S rRNA gene with an approximate amplicon size of 123 bp. For *P. gingivalis*, the PorGin dtec-qPCR Kit (Genetic PCR Solutions™, Spain, cat. no. MGP202) was used, targeting the rgpA gene with an amplicon size of 183 bp. Both kits contain pre-optimized primers and FAM-labelled hydrolysis probes, validated for species-specific detection. Although the exact primer sequences are proprietary and not publicly disclosed, both assays are CE-IVD certified and supported by published validation studies (e.g. [17].

The qPCR reaction for *F. alocis* consisted of 12.5 µl of 2X Master Mix, 5 µl of Oligo Mix (primers and probe), 1–5 µl of DNA template and nuclease-free water to reach a final volume of 25 µl. For *P. gingivalis*, 1 µl of the reconstituted TargetSpecies dtec-qPCR-mix was used in a 20 µl reaction, which also included 9 µl DNase/RNase-free water and 5 µl of GPS™-mix.

Each qPCR run included serial dilutions of a positive control template to generate standard curves for absolute quantification, as well as no-template (negative) controls to confirm the absence of contamination.

Thermal cycling was performed as follows: an initial denaturation step at 95 °C for 2 min, followed by 45 cycles of denaturation at 95 °C for 5 s and annealing/extension at 55 °C for *F. alocis* or 60 °C for *P. gingivalis*, both for 20 s. Fluorescence signals were captured in real time using the FAM channel for the target organisms and the HEX channel for internal control monitoring.

This qPCR protocol was conducted in a closed-tube system, and thus, no gel electrophoresis images were generated. Amplification was assessed through Cq values, melt curve profiles (where applicable), and standard curve analysis. These measures confirmed the efficiency, specificity and reproducibility of the assays.

Conventional microbiological techniques such as culture were not employed in this study. This decision was based on the limitations of culture methods in detecting fastidious and anaerobic organisms such as *P. gingivalis* and *F. alocis*, which are difficult to recover reliably *in vitro*. Real-time PCR provides superior sensitivity and allows precise quantification of target DNA even at low abundance, making it a suitable and widely accepted approach for microbiological analysis in periodontal and peri-implant research.

### Statistical analysis

Statistical analysis was performed using SPSS 26 (IBM Corp., Armonk, NY, USA), with a significance level set at *P*≤0.05 for all tests. Descriptive statistics were calculated for all variables, with continuous variables, such as PD, CAL and *F. alocis* and * P. gingivalis* loads, reported as means±sd, and categorical variables, such as smoking status, presented as frequencies and percentages.

The assumption of normality for continuous variables was evaluated using the Shapiro–Wilk test. Since the data did not follow a normal distribution, non-parametric tests were applied. To compare differences between the healthy/mucositis group and the peri-implantitis group, the Mann–Whitney *U* test was used for continuous variables, while Pearson’s chi-square test was applied to categorical variables.

The correlation between *F. alocis* and *P. gingivalis* levels and clinical parameters, including PD, CAL and BOP, was assessed using Spearman’s rank correlation coefficient (*ρ*). Correlation values were interpreted as weak (0–0.3), moderate (0.3–0.7) or strong (>0.7), with corresponding *P*-values used to determine statistical significance.

## Results

### Demographic characteristics

A total of 110 participants were included in the study, divided into 2 groups: those with peri-implantitis (*n*=52) and those with healthy implants (*n*=58). The demographic characteristics of participants, including age, sex and smoking status, were balanced between the groups. No significant differences were found in terms of age or smoking status between the groups (Table 1).

**Table 1.** Demographic characteristics of the patients

Demographic parameter	Healthy/mucositis (*n*=58)	Peri-implantitis (*n*=52)	Total (*n*=110)
Sex, *n* (%)			
Male	34 (58.6%)	31 (59.6%)	65 (59.1%)
Female	24 (41.4%)	21 (40.4%)	45 (40.9%)
Smoking status, *n* (%)			
Non-smokers	34 (58.6%)	31 (59.6%)	65 (59.1%)
Smokers	24 (41.4%)	21 (40.4%)	45 (40.9%)
Age (years, mean±sd)	54.4±11.63	65.1±8.34	–

### Clinical parameters

Significant differences were observed in the clinical parameters between the healthy/mucositis and peri-implantitis groups. The peri-implantitis group exhibited significantly higher values for PD, CAL and BOP compared to the healthy/mucositis group, as presented in Table 2.

**Table 2.** Comparison of clinical parameters between healthy/mucositis and peri-implantitis groups

Clinical parameter	Healthy/mucositis (mean±sd)	Peri-implantitis (mean±sd)	*P*-value
PD (mm)	3.42±0.86	4.83±1.51	<0.001*
CAL (mm)	3.44±0.90	6.33±2.84	<0.001*
BOP (%)	28.8±34.25	68.63±35.19	<0.001*

PD (mm): The distance from the gingival margin to the base of the pocket. CAL (mm): The measurement indicating the extent of attachment loss. BOP (%): The percentage of sites that bled upon probing. sd: Measure of variability in the data.

*Statistically significant differences were found using the Mann–Whitney *U* test, with significance set at the 0.05 level.

### Microbiological findings

For *F. alocis*, the mean level in the peri-implantitis group was significantly higher (4.58×10^5^±3.40×10^5^ copies µl^−1^) compared to the healthy group (2.45×10^3^±1.64×10^3^ copies µl^−1^), with a *P*-value of less than 0.001.

**Fig. 1.**
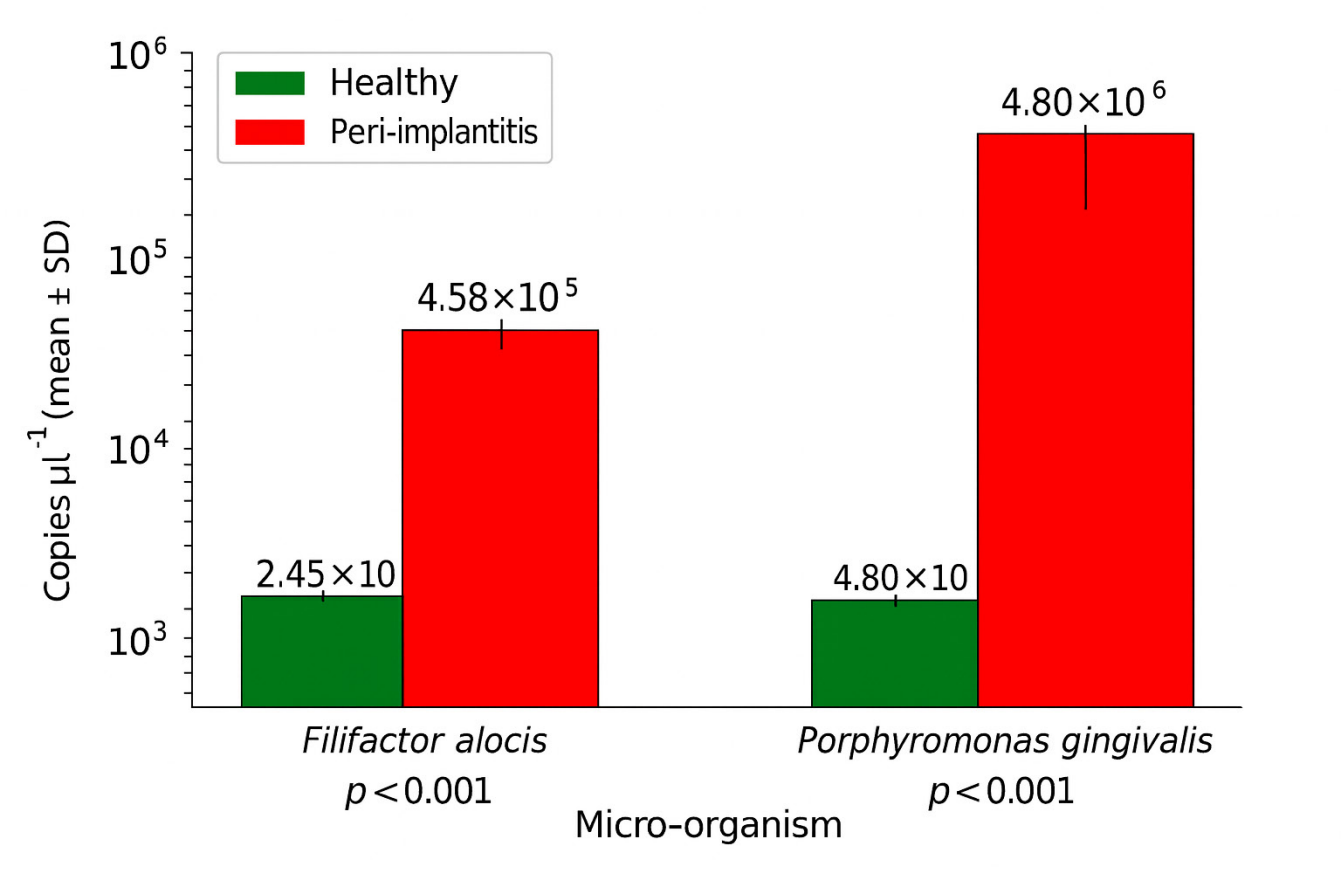
Microbial levels in peri-implant health and disease. Bar plots showing the quantitative levels of key microbial species in peri-implant health, mucositis and peri-implantitis. Statistical comparisons between groups were conducted using the Mann–Whitney *U* test. A *P*-value <0.05 was considered statistically significant.

Similarly, for *P. gingivalis*, the peri-implantitis group had a significantly higher microbial load (4.80×10^6^±4.78×10^6^ copies µl^−1^) compared to the healthy group (2.09×10^3^±1.26×10^3^ copies µl^−1^), also with a *P*-value of less than 0.001 (Fig. 1).

Statistical analysis showed significant differences between the two groups for numbers of both *F. alocis* (Man–Whitney *U*=41.000, *P*<0.001) and *P. gingivalis* (Man–Whitney *U*=29.000, *P*=0.014).

### Correlation analysis between *P. gingivalis*, *F. alocis *and clinical parameters

**Fig. 2.**
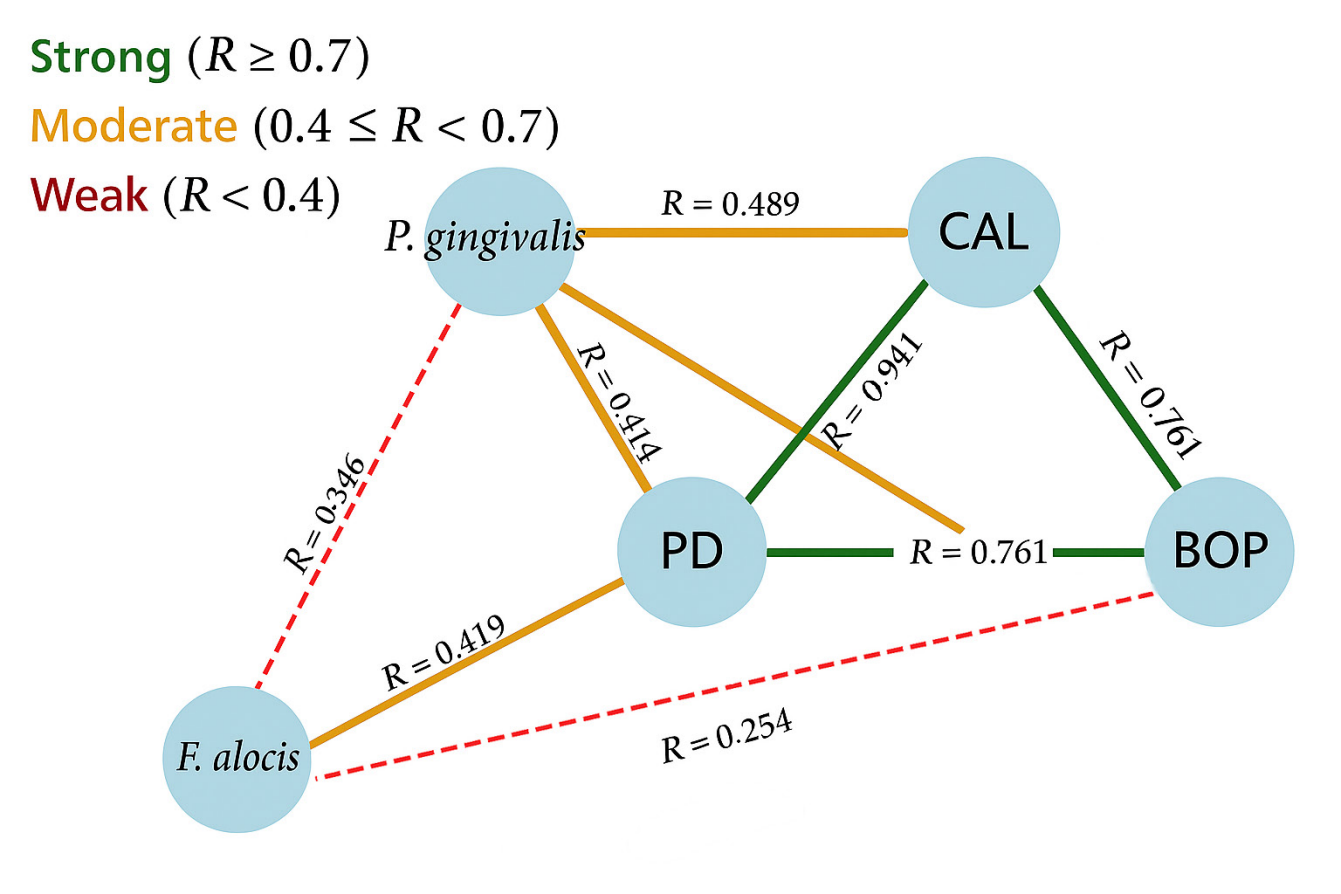
Correlations of microbial and clinical parameters. Scatterplots depicting the relationships between microbial loads and clinical parameters across the study groups. Spearman’s rank correlation coefficients (*ρ*) and corresponding *P*-values are reported for each association.

*P. gingivalis* demonstrated statistically significant correlations with clinical parameters, such as PD (*r*=0.474, *P*=0.019) and CAL (*r*=0.489, *P*=0.015). Additionally, it showed a strong correlation with bleeding on probing (*r*=0.575, *P*=0.003). In contrast, * F. alocis* exhibited statistically significant but moderate correlations with PD (*r*=0.419, *P*=0.017) and CAL (*r*=0.377, *P*=0.033). However, it did not show significant correlations with bleeding on probing (*r*=0.254, *P*=0.161). The correlation between *F. alocis* and *P. gingivalis* was also not statistically significant (*r*=0.346, *P*=0.247). These correlations are illustrated in Fig. 2.

### Impact of smoking on clinical parameters and microbial presence

No significant differences were observed between smokers and non-smokers regarding *F. alocis*, *P. gingivalis*, PD, CAL or BOP. Statistical analysis using the Kruskal–Wallis test indicated no significant associations for *F. alocis* (*H*=1.638, df=2, *P*=0.441), * P. gingivalis* (*H*=0.910, df=2, *P*=0.635), PD (*H*=3.722, df=2, *P*=0.156), CAL (*H*=1.691, df=2, *P*=0.429) or BOP (*H*=0.667, df=2, *P*=0.716). These findings suggest that smoking status did not significantly influence microbial load or clinical parameters in the study population.

## Discussion

The present study aimed to determine the prevalence and levels of *P. gingivalis* and *F. alocis* in peri-implantitis and healthy peri-implant sites using qPCR. This is the first study to use real-time PCR to quantify *F. alocis* in peri-implantitis, providing new insights into the microbial dynamics of peri-implant disease. The results showed significant differences in microbial profiles between peri-implantitis and healthy sites, with higher numbers of *P. gingivalis* and *F. alocis* in diseased sites.

The microbial load of both *P. gingivalis* and *F. alocis* was significantly higher in peri-implantitis compared to healthy sites, reinforcing their role as key contributors to peri-implant inflammation [3,12, 18]. *P. gingivalis*, a member of the ‘red complex’, is well-known for its association with periodontal and peri-implant diseases, driven by its numerous virulence factors, including fimbriae, gingipains and capsules, which allow it to colonize, evade immune responses and contribute to tissue destruction [19]. The significantly elevated presence of *P. gingivalis* in peri-implantitis sites aligns with existing literature, further supporting its involvement in the pathogenesis of peri-implant disease [20].

*P. gingivalis* contributes to peri-implant tissue destruction through a range of well-characterized virulence mechanisms. These include fimbriae that mediate adhesion and invasion, capsules that protect it from phagocytosis and gingipains (cysteine proteases) that degrade extracellular matrix proteins and host cytokines [15]. Furthermore, *P. gingivalis* is known for immune subversion; it interferes with the complement system through C5a receptor–toll-like receptor 2 (TLR2) cross-talk and inhibits neutrophil clearance, thereby promoting a dysbiotic inflammatory environment [15,19]. Its keystone pathogen behaviour disrupts microbial homeostasis, allowing pathogenic consortia to flourish and sustain chronic inflammation.

*F. alocis*, although less extensively studied, has emerged as a critical organism in both periodontitis and peri-implantitis. It exhibits notable resistance to oxidative stress and thrives in inflammatory conditions. Mechanistically, *F. alocis* activates TLR2-mediated pathways and induces pro-inflammatory cytokines, such as IL-6 and IL-8, in gingival fibroblasts. It also enhances the pathogenicity of *P. gingivalis* when co-cultured by increasing biofilm mass and immune evasion capacity [12,13]. Moreover, it modulates neutrophil function and prolongs inflammation by preventing resolution of the innate immune response.

The role of *F. alocis* in peri-implantitis, however, is less investigated in the literature, and previous studies have largely focused on *F. alocis* in the context of periodontal disease [12,21]. In the present study, *F. alocis* was found in statistically significantly higher numbers in peri-implantitis sites, highlighting its emerging role as a possible pathogen in both peri-implant and periodontal diseases. The current study is among the first to use qPCR aiming to quantify *F. alocis* in peri-implantitis, thus contributing to our understanding of peri-implant microbiology.

Present data exhibited statistically significant positive correlations with clinical parameters such as PD and CAL for both investigated species, while *F. alocis* failed to demonstrate this correlation for bleeding on probing. This finding might suggest that while *P. gingivalis* is closely associated with clinical disease severity, *F. alocis* may play a more complex, indirect role in modulating the peri-implant environment, contributing to biofilm persistence and immune modulation rather than directly influencing clinical parameters. The weak statistical correlation between numbers of *P. gingivalis* and *F. alocis* observed in the present study also suggests that while they co-exist and enhance each other’s pathogenic potential, they may contribute to the disease through distinct mechanisms.

While this study focused on *P. gingivalis* and *F. alocis*, the results should be interpreted in light of the broader complexity of the peri-implant microbiota. In addition to *P. gingivalis* and *F. alocis*, other pathogens have been implicated in peri-implantitis. These include anaerobes like *Tannerella forsythia*, *Treponema denticola*, *Fusobacterium nucleatum, Prevotella intermedia* and *Campylobacter rectus,* which may act synergistically to promote inflammation and bone loss [9,22]. Opportunistic organisms, such as *Staphylococcus epidermidis* and *Candida albicans*, and even viruses like herpesviruses have also been detected in peri-implant lesions, particularly in advanced or refractory cases [23]. Peri-implantitis and mucositis are associated with shifts in the microbial ecosystem that involve multiple bacterial and fungal species, as well as viruses. Future studies utilizing metagenomic sequencing and broader microbial profiling could help elucidate how these organisms interact and contribute to disease progression. The findings from this study contribute to understanding the roles of *P. gingivalis* and *F. alocis* but underscore the need to see these pathogens within the larger microbiological context of dysbiosis in peri-implant environments.

Our findings are consistent with studies that describe the microbial profile of peri-implantitis as being characterized by an increased presence of species such as *P. gingivalis* and *F. alocis* [8,20, 22]. Sanz-Martin et al. [24] and Kensara et al. [23] have also reported on the increased prevalence of *P. gingivalis* in peri-implantitis, but *F. alocis* was either not investigated or its role was not well characterized in peri-implant studies [23,24]. This study, therefore, contributes to the growing understanding of * F. alocis* in peri-implantitis, alongside the well-established role of *P. gingivalis*.

Interestingly, smoking did not appear to have a significant impact on microbial loads or clinical parameters in this study. While smoking is a recognized risk factor for peri-implant disease, this finding may reflect the specific characteristics of the study population, such as the relatively small number of smokers or variability in smoking intensity. Larger, stratified cohorts may be needed to clarify the role of smoking in peri-implant microbial dynamics and clinical outcomes.

The cross-sectional design of this study does not allow for establishing causal relationships between microbial shifts and disease progression. Longitudinal studies are needed to better understand the temporal dynamics of microbial changes and their direct impact on peri-implant health and disease. The use of real-time PCR in this study provided key advantages for assessing microbial loads of investigated species. Real-time PCR’s closed system also reduced contamination risk compared to conventional PCR, enhancing data reliability. However, qPCR’s limitation lies in its inability to analyse the broader microbiome, which future studies using next-generation sequencing could address.

More advanced molecular techniques, such as next-generation sequencing, could provide a more comprehensive profile of the peri-implant microbiome, offering insights into the bacterial composition and their functional contributions to disease. Metagenomic approaches could further elucidate the interactions and metabolic pathways that underpin the pathogenic synergy between *P. gingivalis* and *F. alocis*, ultimately guiding more effective therapeutic interventions [23].

In addition, it is crucial to note that despite its association with peri-implantitis, as observed in the current study, there is not yet conclusive evidence that *F. alocis* is a pathogenic organism directly causing disease. Its presence may indicate dysbiosis, but it remains uncertain whether it plays an active role in pathogenesis or is simply a bystander within a disturbed microbial environment. This distinction is critical in evaluating its role in peri-implantitis, as the mere presence of *F. alocis* does not necessarily confirm its pathogenicity.

The findings of the present study have several clinical implications. The significantly higher prevalence of *P. gingivalis* and * F. alocis* in peri-implantitis sites indicates that these pathogens may require targeted antimicrobial strategies to reduce their levels specifically. For instance, antimicrobials that disrupt biofilm formation or selectively target *P. gingivalis* and *F. alocis* could be beneficial in managing peri-implantitis and preventing further progression. Early identification of *F. alocis* could enable more tailored and effective treatment strategies to halt the progression of peri-implant disease. The association between the presence or counts of *P. gingivalis* and *F. alocis* and a history of previously treated periodontal disease warrants further consideration. Notably, the study successfully recruited many individuals who were periodontally healthy in this age group, potentially reflecting stringent inclusion criteria or targeted recruitment strategies. However, future studies could explore whether residual effects of previously treated periodontal disease might influence peri-implant microbial profiles, even in individuals classified as periodontally healthy.

In conclusion, the present study highlights the significant role of *P. gingivalis* and *F. alocis* in peri-implantitis. The possible synergistic relationship between these pathogens enhances the pathogenicity of the peri-implant biofilm, underscoring the need for targeted therapeutic strategies that disrupt these microbial interactions. Future research should focus on developing novel antimicrobial therapies to reduce the pathogenic burden of these key bacteria and restore the microbial homeostasis necessary for peri-implant health.

## Clinical trial registration

This study is registered at ClinicalTrials.gov under ID: NCT05711407.

## Abbreviations

BOP: bleeding on probing
CAL: clinical attachment level
PD: probing depth
qPCR: quantitative real-time PCR
TLR2: toll-like receptor 2
